# Major depression mistaken as frontotemporal dementia due to PET scan

**DOI:** 10.1177/20542704241241113

**Published:** 2024-04-02

**Authors:** Martin Bystad, Ole Grønli, Rolf Wynn

**Affiliations:** 1 Division of Substance Use and Mental Health, 60519 University Hospital of North Norway, Tromsø, Norway; 2 Department of Clinical Medicine, 8016 UiT The Arctic University of Tromsø, Tromsø, Norway; 3 Department of Education, ICT and Learning, 3678 Østfold University College, Halden, Norway

**Keywords:** Frontotemporal dementia, PET, neuroimage, depression, neuropsychology

## Abstract

Clinicians should be aware that the hypometabolism associated with depression can mimic frontotemporal dementia on PET.

Frontotemporal dementia (FTD) is associated with progressive degeneration of the frontal lobes and this leads to changes in language, motor symptoms, behavior and executive functions.^1^ In an early stage, patients with FTD usually have intact memory functions.^2^ 40% of the cases of FTD are misdiagnosed,^3^ with delayed diagnosis compared to other dementias.^4^ Differentiating FTD from other psychiatric disorders poses challenges, given executive impairment is a common symptom across disorders.^5^ The need for diagnostic tools has led to the increased use of positron emission tomography (PET), which is regarded as the most accurate in-vivo method for investigating brain metabolism.^6^ We present a case where PET was central to the diagnostic process.

## Case report

A 67-year-old Caucasian male with no prior psychiatric history was admitted to psychiatric hospital three times during one year. Except for bilateral coxarthrosis, he had no previous history of somatic disease. The patient had higher education and was supported by family and close friends. Two months before the first admission, he was confused and apathetic. His general practitioner prescribed the antidepressant mirtazapine, 30 mg once daily, and the antipsychotic risperidone, 0.5 mg once daily, without any obvious effect. He displayed increasing restlessness, irritability, poor judgement, confusion and apathy, and he was admitted to psychiatric hospital. During this first admission, he could wander around for hours without any clear purpose and it was difficult for him to have an ordinary conversation. A Montgomery-Aasberg Depression Rating Scale (MADRS) interview was conducted and suggested a moderate depression. However, based on observation of the patient's behaviour and on an overall clinical assessment, a more severe psychotic depression was suspected according to the criteria of the International Classification of Diseases, tenth revision (ICD-10), and he was therefore treated with the antipsychotic olanzapine 10 mg daily and the mood stabilizer lithium 42 mg daily. The patient showed some improvement and he was discharged and the medication continued. However, his condition worsened quickly, and he was readmitted for a second hospital stay only four weeks after his first discharge.

During his second admission, he displayed symptoms of apraxia, difficulties with coordination, he walked slowly, and could not perform diagonal movements. It was suspected that these symptoms represented side effects, and the motor symptoms faded when olanzapine was terminated.

Staff also observed that the patient's face appeared expressionless. Thus, Parkinson’s disease was suspected. Neither a clinical neurological examination nor a Dopamine Transporter Scan suggested Parkinson's disease. As the patient did not have a typical clinical picture nor any obvious memory decline, Alzheimer's disease was not considered likely. A cerebrospinal fluid sample revealed that all relevant markers, including T-tau, P-tau and beta-amyloid, were within the normal range.

His confusion and apathy improved, and he was discharged. However, also this remission was quite brief, and he developed an increasing lack of initiative, and worsening confusion and apathy. He denied symptoms of sadness, anhedonia, grief or hopelessness. Three months after his second hospital stay, he was readmitted with a suspected depressive disorder.

A lack of response to prior medication attempts prompted a shift in treatment to bilateral electroconvulsive therapy (ECT), which, despite providing some relief, did not fully address apathy and poor judgment after 14 sessions. MADRS results did not suggest an ongoing depression at this stage.

The patient’s family described that his personality had changed over the last few years and that he had developed difficulties with verbal expression, apraxia, decision-making, planning, initiative and apathy. A neurodegenerative disease was suspected. A neuropsychological assessment two weeks after the last ECT session gave scores within normal range on all neuropsychological tests.

**Figure 1.**
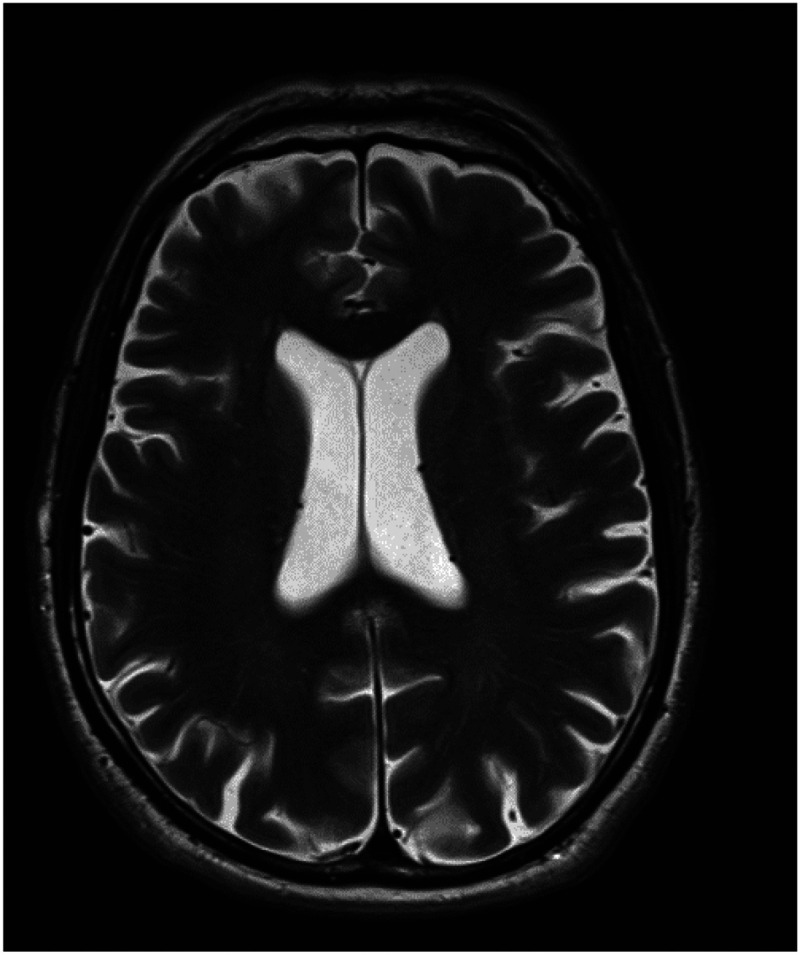
PET scan.

A screening questionnaire (Frontal Behavior Inventory) yielded scores slightly above the cut-off for FTD. The patient's symptoms, and especially his verbal difficulties, apraxia, judgement and apathy, were judged to be compatible with an early stage of FTD. Magnetic Resonance Imaging was performed once without any signs of past or present stroke, dementia, intracranial mass, or any other pathological findings. A flour-18-labelled fluorodeoxyglucose (18 FDG-PET) scan revealed distinct frontal lobe hypometabolism, which was judged compatible with FTD, specifically primary progressive aphasia (Figure 1). Subsequently, the patient was diagnosed with FTD and discharged.

Twelve months later, a follow-up psychiatric assessment revealed considerable improvement in apathy and judgement. The family now described his functioning as normal and were no longer worried about his behavior. The family stated that the apathy and ability to make decisions had improved substantially, and there were no difficulties with having a conversation nor any irritability. The clinical picture at this stage was not compatible with FTD or any other type of dementia. Neuropsychological tests yielded normal results. A follow-up PET scan revealed no sign of hypometabolism.

Upon reviewing the patient's medical history and diagnostic process, including the exclusion of other diagnoses, the most likely conclusion was a depressive disorder with an atypical presentation, mistaken for FTD.

## Discussion

This case study suggested that the initial PET scanning failed to detect the patient's depression and that he was misdiagnosed with FTD. A major depression (with psychotic symptoms) was initially suspected. However, the patient's clinical development and poor response to treatment as well as the initial PET findings weakened the depression hypothesis. A bipolar disorder was considered less likely, since the patient had no history of mania or prior depression. The patient did not have any underlying somatic conditions nor did he fulfill the diagnostic criteria for delirium.

Both depression and FTD present with frontal hypometabolism. The sensitivity of PET for the detection of frontal dementia is  > 90%,^7^ but a lower specificity (68–74.6%) may increase the chances of an erroneous diagnosis of dementia, as in our case.

PET is generally recommended as a diagnostic tool for FTD.^8^ Clinicians should be aware that the hypometabolism associated with depression can mimic FTD on PET. The incorrect dementia diagnosis in our case was a severe burden on both the patient and his family.

Executive functions may be impaired in patients suffering from a range of psychiatric diseases, including FTD.^9^ When there is a suspicion of FTD, applying neuropsychological tests with higher specificity, for instance Iowa Gambling Task and Wisconsin Card Sorting Test, may be helpful.^10^

While our study includes only one case, it is a reminder that PET scanning should be an additive rather than a stand-alone diagnostic tool. To our knowledge, no studies have systematically investigated how PET can be applied as an aid to distinguish depression from FTD and this should be explored in future studies.

## Conclusions

Clinicians should be aware that the hypometabolism associated with depression can mimic FTD on PET.
